# 全身麻醉及现场快速评价在EBUS-TBNA诊断肺癌中的价值

**DOI:** 10.3779/j.issn.1009-3419.2024.102.07

**Published:** 2024-02-20

**Authors:** Yuhe HU, Yuying LI

**Affiliations:** 646000 泸州，西南医科大学附属医院呼吸与危重症医学科; Department of Respiratory and Critical Care Medicine, The Affiliated Hospital of Southwest Medical University, Luzhou 646000, China

**Keywords:** 肺肿瘤, 超声引导下经支气管针吸活检, 全身麻醉, 快速现场评价, Lung neoplasms, Endobronchial ultrasound-guided transbronchial needle aspiration, General anesthesia, Rapid on-site evaluation

## Abstract

**背景与目的** 肺癌是常见的呼吸系统恶性肿瘤。超声引导下经支气管针吸活检（endobronchial ultrasound-guided transbronchial needle aspiration, EBUS-TBNA）是诊断肺癌和评估分期的重要工具。EBUS-TBNA大多在局部麻醉或清醒镇静下进行，而在全身麻醉下行EBUS-TBNA以及同时应用快速现场评价（rapid on-site evaluation, ROSE）能否进一步提高诊断效能目前仍未可知，本研究拟探索全身麻醉及ROSE在EBUS-TBNA诊断肺癌中的价值。**方法** 回顾性分析164例于2018年1月至2022年12月于西南医科大学附属医院呼吸与危重医学科就诊患者的资料，所有患者术前均疑诊为肺癌并行EBUS-TBNA，根据是否行全身麻醉及ROSE将患者分为局麻组（n=54）、全麻组（n=67）和全麻ROSE组（n=43），分析各组的穿刺情况以及在疾病诊断方面的差异。**结果** 局麻组的淋巴结穿刺针数高于全麻ROSE组（P<0.01）。三组患者的疾病总诊断率分别为87.04%、89.55%和90.70%，恶性肿瘤的诊断率分别为88.24%、88.89%和94.74%，均无统计学差异（P>0.05）。三组中没有患者出现严重并发症及麻醉相关不良反应。**结论** 与局部麻醉联合静脉镇痛镇静相比，全身麻醉下实施EBUS-TBNA无论是否联合ROSE均可得到同样准确的结果，全身麻醉联合ROSE可以减少淋巴结穿刺针数。

## Abstract (en)

**Background and objective** Lung cancer is a common malignant tumor of respiratory system. Endobronchial ultrasound-guided transbronchial needle aspiration (EBUS-TBNA) is a valuable tool for the diagnosis and staging of lung cancer. EBUS-TBNA is predominantly performed under local anesthesia or conscious sedation. However, the diagnostic performance of EBUS-TBNA under general anesthesia and in conjunction with rapid on-site evaluation (ROSE) remains uncertain. This study aims to investigate the value of general anesthesia and ROSE in the diagnosis of lung cancer with EBUS-TBNA. **Methods** A retrospective analysis was conducted on 164 patients treated in the Department of Respiratory and Critical Care Medicine of The Affiliated Hospital of Southwest Medical University from January 2018 to December 2022. All patients were preoperatively suspected of lung cancer and underwent EBUS-TBNA. Based on whether they received general anesthesia and ROSE, the patients were divided into three groups: local anesthesia group (LA group)(n=54), general anesthesia group (GA group)(n=67) and general anesthesia with ROSE group (GA-ROSE group)(n=43). The puncture characteristics and diagnostic differences were analyzed among the groups. **Results** The number of lymph node puncture needles in the LA group was higher than in GA-ROSE group (P<0.01). The overall diagnostic rates of EBUS-TBNA for the three groups were 87.04%, 89.55% and 90.70%, respectively, with malignant tumor diagnostic rates of 88.24%, 88.89% and 94.74%. No statistically significant differences were observed among the three groups (P>0.05). There were no instances of severe complications or adverse anesthesia reactions in any of the groups. **Conclusion** Compared to the combination of local anesthesia with intravenous analgesia and sedation, the implementation of EBUS-TBNA under general anesthesia, with or without ROSE, achieves equally accurate results, and general anesthesia combined with ROSE can reduce in the number of lymph node puncture needles.

肺癌是发病率和死亡率最高的恶性肿瘤之一^[1]^，随着技术的进步，诊断方法也日趋多样。过去10余年里，超声引导下经支气管针吸活检（endobronchial ultrasound-guided transbronchial needle aspiration, EBUS-TBNA）已成为肺癌评估的重要工具，在肺癌诊断及分期方面具有较高的敏感性和特异性^[2,3]^。过去，EBUS-TBNA多在局部麻醉和清醒镇静下进行，有研究^[4]^表明，在全身麻醉下行EBUS-TBNA可以增加淋巴结标本取样量、改善患者舒适度、提高诊断效能，但也有研究^[5,6]^报道，无论是在清醒镇静还是全身麻醉下行EBUS-TBNA，诊断效能及并发症并无明显差异。目前，尚无研究或指南推荐哪种麻醉方法具有绝对优势。

快速现场评价（rapid on-site evaluation, ROSE）应用于介入呼吸病学开始于20世纪80年代，Pak等^[7]^将肺部病灶穿刺针吸标本进行床旁快速染色，5 min内完成，15 min内得到结果。随着EBUS-TBNA在国内的广泛应用，对穿刺样本诊断成功率提出了更高的要求，肺癌的介入诊断朝着精确化、微创化发展，ROSE所具有的快速、高效的特点使之在介入诊断领域有了越来越高的地位。研究表明，在EBUS-TBNA期间联合ROSE可以提高诊断效能^[8⇓-10]^、减少穿刺针数^[11,12]^、减少并发症^[13]^，而麻醉方法对EBUS-TBNA诊断肺癌有无影响、全身麻醉联合ROSE能否在原有基础上提高诊断效能尚不得而知，国内外亦无相关研究报道，因此，本研究将立足于临床实践，探讨全身麻醉及ROSE在EBUS-TBNA诊断肺癌中的价值。

## 1 资料与方法

### 1.1 研究人群

本研究收集了2018年1月至2022年12月至西南医科大学附属医院呼吸与危重医学科就诊并行EBUS-TBNA患者的资料。纳入标准：（1）术前行胸部计算机断层扫描（computed tomography, CT）平扫或增强扫描提示肺内病灶伴或不伴纵隔、肺门淋巴结增大（淋巴结短径>1 cm），结合临床资料怀疑肺恶性肿瘤者；（2）病历资料完整。收集患者年龄、性别、吸烟史等基本信息以及病灶及淋巴结情况、穿刺情况、病理结果、最终诊断等资料。并根据患者是否行全身麻醉及ROSE将其分为三组，分别为局麻组、全麻组和全麻ROSE组。本研究得到了西南医科大学附属医院伦理委员会批准（批准号：KY2023314）。

### 1.2 操作方法

#### 1.2.1 麻醉方法

局麻组使用利多卡因雾化口咽部和静脉使用镇痛镇静药物，静脉药物为芬太尼与咪达唑仑联合使用，保留自主呼吸，超声支气管镜经鼻进入气道，术中根据患者情况决定是否追加药物；全麻组及全麻ROSE组使用全身麻醉，在此期间患者意识丧失，即使受到疼痛刺激也不能唤醒，无自主呼吸。麻醉诱导后插入喉罩，通过麻醉机控制通气，超声支气管镜经喉罩进入气道，术中可能使用到的药物包括异丙酚、芬太尼、氯胺酮、舒芬太尼、依托咪酯和顺阿曲库铵。

#### 1.2.2 EBUS-TBNA操作过程

首先对所有患者行常规支气管镜检查（Olympus BF-260）观察气道内情况，随后换用超声支气管镜（Olympus UC-260-FW，超声主机为Olympus EU-ME1）行EBUS-TBNA。使用生理盐水膨胀超声探头水囊，在患者胸部CT提示有肺内病灶或纵隔、肺门有异常增大淋巴结处进行探查，调节探头使之处于病灶或淋巴结的最大短径处，打开彩色多普勒确定病灶内及周围血供情况，测量病灶大小，避开血管，选择合适的穿刺点，通过工作孔道送入穿刺针（Olympus NA-201SX-4021, 21 G）并固定于超声支气管镜上，调整外鞘管，确认穿刺点后刺入病灶，拔除针芯后连接负压吸引器，反复抽吸穿刺20-30次，穿刺完成后移除负压吸引器、拔出穿刺针，对穿刺标本进行处理。

局麻组及全麻组所获得的组织条标本经福尔马林固定后送病理组织学检查，由病理科医师进行读片并根据样本情况决定是否行免疫组化，同时将穿刺所获得的细胞学涂片经95%乙醇固定后送细胞学室进行细胞病理学检查。全麻ROSE组患者每次穿刺后将由细胞学医师进行现场制片，采用目前所公认的迪夫快速染色法进行染色，根据显微镜下的细胞学结果进行快速现场判读，若结果提示明确的定性诊断（如查见癌细胞）或查见较多淋巴细胞，认为标本基本合格；若结果提示仅查见血细胞、上皮细胞、坏死样物质，则认为标本不合格。当ROSE结果为查见癌细胞且细胞数量充足时，考虑可在原位置继续穿刺；当ROSE结果认为标本不合格或标本合格但未查见癌细胞或癌细胞数量较少时，将更换位置进行穿刺，具体穿刺针数由术者决定，但每个病灶的穿刺针数不超过4次^[14]^。与局麻组及全麻组相同，全麻ROSE组所获得的组织条标本和细胞学涂片将分别送病理组织学检查及细胞病理学检查。

### 1.3 诊断标准

当EBUS-TBNA所获得的组织条标本或细胞学标本得到明确的病理诊断结果，则以此为最终诊断；当组织条标本或细胞学标本不能得到明确的病理结果，而患者通过其他途径如外科手术、淋巴结活检、肺穿刺、胸腔镜或二次检查获得了明确的病理组织学结果，则以该途径所得病理诊断结果为最终诊断；若EBUS-TBNA所得标本未得到明确病理诊断结果且患者当时未经其他途径进一步明确诊断时，则影像学随访至少半年后以当时临床诊断为最终诊断。

### 1.4 统计学方法

数据分析采用SPSS 26.0。计量资料采用均数±标准差表示，三组间比较采用单因素方差分析，两两比较采用Scheffe法（方差齐）和Games-Howell法（方差不齐）；计数资料采用例数（百分比）表示，组间比较采用卡方检验。采用双侧检验，P<0.05为差异有统计学意义。

## 2 结果

### 2.1 基本情况

所有患者基本情况如表1所示。共164例患者纳入该研究，其中男性124例，女性40例。局麻组54例，全麻组67例，全麻ROSE组43例。三组在年龄、性别方面没有统计学差异。行EBUS-TBNA后发现，三组患者的穿刺肿块直径、穿刺肿块针数、穿刺淋巴结短径没有统计学差异，而在穿刺淋巴结针数方面，局麻组的穿刺针数明显高于其他两组，且局麻组和全麻ROSE组的穿刺针数有统计学差异（P<0.01）。

**表1:** 三组患者的一般情况及穿刺情况

Characteristics	LA group (n=54)	GA group (n=67)	GA-ROSE group (n=43)	P
Age (yr)	54.69±8.72	61.61±9.95	58.72±11.57	0.09
Gender				0.17
Male	36 (66.67%)	53 (79.10%)	35 (81.40%)	
Female	18 (33.33%)	14 (20.90%)	8 (18.60%)	
Smoking status				0.03
Current smoker	26 (48.15%)	47 (70.15%)	28 (65.12%)	
Former smoker	4 (7.41%)	3 (4.48%)	6 (13.95%)	
Never-smoker	24 (44.44%)	17 (25.37%)	9 (20.93%)	
Mass diameter (cm)	4.50±1.29	3.80±1.20	4.00±1.41	0.60
Puncture number of mass	3.24±1.17	3.37±0.87	3.71±0.66	0.54
LN diameter (short axis) (cm)	2.18±0.63	2.36±1.03	2.29±0.78	0.46
Puncture number of LN	3.58±1.61	2.98±1.33	2.57±1.13**	<0.01

**: Compared with LA group, P<0.01. LA: local anesthesia; GA: general anesthesia; GA-ROSE: general anesthesia with ROSE; LN: lymph node.

### 2.2 穿刺情况

三组患者肺内病灶及淋巴结穿刺情况如表2所示。一共进行了197次肺内病灶及淋巴结穿刺。其中局麻组有4处肺内病灶、64处淋巴结穿刺。全麻组有20处肺内病灶、58处淋巴结穿刺，全麻ROSE组有9处肺内病灶、42处淋巴结穿刺。在所有淋巴结穿刺中，4R组及7组淋巴结进行的穿刺最多。

**表2:** 三组患者穿刺情况分布

Index	LA group (n=54)	GA group (n=67)	GA-ROSE group (n=43)
Mass	4	20	9
Lymph node stations			
2R	0	2	0
2L	0	0	0
4R	21	19	11
4L	0	1	3
7	34	30	23
10R	1	0	0
10L	1	2	0
11Rs	3	0	2
11Ri	0	1	2
11L	4	2	1
12R	0	0	0
12L	0	1	0
Total	68	78	51

### 2.3 EBUS-TBNA诊断情况

如表3所示。共有146例患者通过EBUS-TBNA得到了明确诊断，其中局麻组有47例（包括10例鳞癌、21例腺癌、9例小细胞癌、1例大细胞癌、4例肺外恶性肿瘤、2例良性疾病）；全麻组有60例（包括10例鳞癌、25例腺癌、19例小细胞癌、2例肺外恶性肿瘤、4例良性疾病）；全麻ROSE组有39例（包括6例鳞癌、16例腺癌、11例小细胞癌、3例肺外恶性肿瘤、3例良性疾病）。

**表3:** 三组患者的诊断情况

Index	LA group (n=54)	GA group (n=67)	GA-ROSE group (n=43)
Confirmed by EBUS-TBNA (n=146)			
Pathological type of the cancer			
Squamous cell carcinoma	10	10	6
Adenocarcinoma	21	25	16
Small cell lung cancer	9	19	11
Large cell lung cancer	1	0	0
Other cancer	4	2	3
Inflammation/infection			
Tuberculosis	2	2	1
Lymph node hyperplasia	0	2	1
Lung abscess	0	0	1
Total	47	60	39
Not confirmed by EBUS-TBNA (n=18)			
Pathological type of the cancer			
Squamous cell carcinoma	1	2	0
Adenocarcinoma	3	4	1
Small cell lung cancer	1	0	0
Large cell lung cancer	0	1	0
Other cancer	1	0	1
Inflammation/infection			
Lung abscess	1	0	0
Tuberculosis	0	0	2
Total	7	7	4

EBUS-TBNA: endobronchial ultrasound-guided transbronchial needle aspiration.

共有18例患者未能通过EBUS-TBNA确诊。局麻组有7例，其中5例通过其他有创检查确诊（包括3例肺腺癌、1例肺脓肿、1例乳腺癌肺转移），2例通过二次检查确诊（包括1例肺鳞癌、1例小细胞癌）；全麻组有7例，其中5例通过其他有创检查确诊（包括3例肺腺癌、1例肺鳞癌、1例大细胞神经内分泌癌），2例通过二次检查确诊（包括1例肺腺癌、1例肺鳞癌）；而在全麻ROSE组中仅有4例，其中2例患者通过经验性治疗、动态随访确诊为肺结核，另外2例通过其他有创检查确诊（包括1例肺腺癌、1例恶性胸腺瘤）。

在诊断率方面，三组患者疾病总体诊断率如图1所示，局麻组和全麻组的总体诊断率分别为87.04%和89.55%，而全麻ROSE组的总体诊断率最高，为90.70%，但三组间的总体诊断率没有表现出统计学差异（P=0.84）。三组患者恶性肿瘤诊断率如图2所示，局麻组和全麻组的诊断率相近，分别为88.24%和88.89%，而全麻ROSE组的恶性肿瘤诊断率最高，为94.74%，但诊断率在三组之间同样没有表现出统计学差异（P=0.54）。

**图1:**
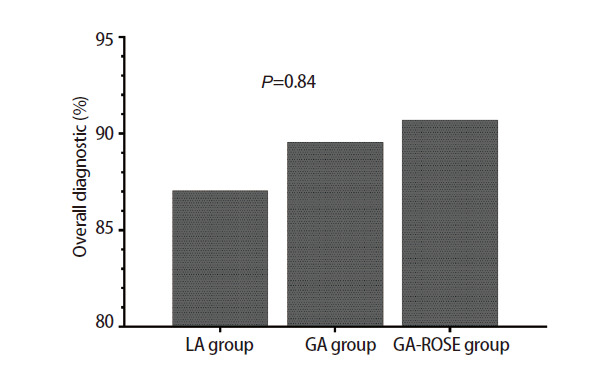
三组患者疾病总体诊断率

**图2:**
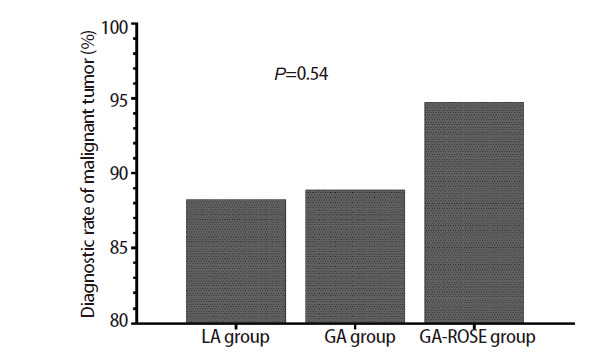
三组患者恶性肿瘤诊断率

### 2.4 EBUS-TBNA并发症

三组中共有5例患者出血较多需要额外的药物处理（稀释肾上腺素、注射用血凝酶、垂体后叶素局部或静脉使用），其中局麻组1例，全麻组3例，全麻ROSE组1例，需要外科手术或介入治疗干预的出血，术后没有患者出现气胸、纵隔气肿、需要行机械通气的低氧血症、死亡以及麻醉相关不良反应等其他严重并发症。

## 3 讨论

作为一种高发病率、高死亡率的恶性肿瘤，肺癌常经淋巴结转移，其肿瘤原发灶-淋巴结-转移（tumor-node-metastasis, TNM）分期尤其是N分期往往决定了患者的治疗方式和手术方式。EBUS-TBNA自2008年引入我国以来得到了长足的发展，成为诊断不明原因纵隔或肺门淋巴结肿大的一线手段，因其具有准确率高、创伤小、简便快速的特点而深受一线临床医师的青睐。

EBUS-TBNA在国内外很多医学中心门诊常规开展，局部麻醉联合静脉镇痛镇静因为操作简洁、费用低成为了门诊患者首选的麻醉方式，而缺点则是部分患者的咳嗽难以控制、无法耐受检查，这在一定程度上影响了检查效果、延长了操作时间。而在全身麻醉下，患者没有痛感、意识丧失，气道始终为开放状态，自主呼吸和咳嗽受到明显抑制，使术者的操作更为流畅，如在术中出现大出血等并发症，也更利于进行抢救。在我们的研究中，局麻组的淋巴结穿刺针数明显高于另外两组，这可能给患者带来了潜在风险。而对诊断结果的影响方面，Yarmus等^[4]^所报道的包括309例患者的回顾性分析表明，相比于使用芬太尼和咪达唑仑进行的中度镇静，在EBUS-TBNA中使用异丙酚进行深度镇静可以提高诊断效能。但Casal等^[5]^在之后开展的随机对照研究显示，在全身麻醉下行EBUS-TBNA的诊断效能、主要并发症与中度静脉镇静相当，之后更多的研究^[6,15]^也认为麻醉方式对诊断结局没有影响，这与本研究的结果基本一致，尽管另外两组的疾病总体诊断率均高于局麻组，但没有出现统计学差异。

相比于在超声下根据病灶情况进行经验性穿刺，ROSE的出现无疑为穿刺病灶的选择提供了更加明确的方向。和麻醉方式带来的影响一样，临床医师最为关心的是这样的技术能否为诊断带来获益。3项关于中国患者的回顾性研究^[8⇓-10]^均显示，EBUS-TBNA联合ROSE可以提高诊断效能、减少穿刺针数，而另外两项关于EBUS-TBNA所得样本行分子检测的研究^[11,16]^表明，肺癌患者在穿刺期间联合ROSE避免了一部分重复进行的侵入性诊断程序、减少了穿刺针数，而Chest上发表的一项综合多个研究的meta分析^[12]^则认为，在TBNA中联合ROSE既不能提高诊断效能，也不能减少穿刺时间，但在EBUS-TBNA中使用ROSE可以减少穿刺次数。而另一项研究^[17]^认为，使用EBUS-TBNA进行标准化的纵隔分期时，ROSE不会影响诊断率或对临床决策有所帮助。综上所述，大多数研究认为EBUS-TBNA联合ROSE可以减少穿刺针数，而是否可以带来诊断效能方面的获益则结论不一。目前尚无对麻醉方式联合ROSE对EBUS-TBNA诊断效能影响进行综合分析的研究。在本研究中，我们发现全麻ROSE组在恶性肿瘤诊断率及疾病总诊断率方面均优于其他两组，全麻ROSE组中未明确诊断的病例数最少，尽管这种差异没有统计学意义，但这可能是由于本研究的样本量相对较小所致。另外，全身麻醉可以改善患者舒适度，相比于全麻ROSE组，局麻组患者淋巴结穿刺次数明显增加，因此，相比于局部麻醉联合静脉镇痛镇静，全身麻醉联合ROSE仍有可能为患者带来诊断效能及其他方面的临床获益。在并发症方面，仅有5例患者因出血较多需要额外的临床干预，没有患者出现严重并发症及麻醉相关不良反应，这表明在EBUS-TBNA过程中采用全身麻醉总体上是安全的。

本研究也有一定的局限性。首先，本研究是一项回顾性分析，纳入病例偏少，局麻组中男女比例和其他两组有所差异，这可能在一定程度上影响了研究结果。此外，因为客观资料的缺乏，我们没有对3组患者进行更为全面、深入的分析，关于全身麻醉及ROSE能否为患者带来更多的临床获益，这有待于以后的研究来进一步证实。

综上所述，在全身麻醉下实施EBUS-TBNA是舒适、安全的，与局部麻醉联合静脉镇痛镇静相比，全身麻醉无论是否联合ROSE均可得到同样准确的结果，全身麻醉联合ROSE可以减少淋巴结穿刺针数。

**Competing interests**

The authors declare that they have no competing interests.

**Author contributions**

Hu YH and Li YY conceived and designed the study. Hu YH performed the experiments, analyzed the data and contributed analysis tools. Hu YH and Li YY provided critical inputs on design, analysis, and interpretation of the study. Both the authors had access to the data, read and approved the final manuscript as submitted.
